# Targeting the plasma membrane of neoplastic cells through alkylation: a novel approach to cancer chemotherapy

**DOI:** 10.1007/s10637-015-0263-1

**Published:** 2015-06-23

**Authors:** Matthew Trendowski, Thomas P. Fondy

**Affiliations:** Department of Biology, Syracuse University, 107 College Place, Syracuse, NY 13244 USA

**Keywords:** Plasma membrane, Alkylating agents, Leukocyte influx, Carbohydrate analogs, Dihydroxyacetone phosphate inhibitors

## Abstract

*Background* Although DNA-directed alkylating agents and related compounds have been a mainstay in chemotherapeutic protocols due to their ability to readily interfere with the rapid mitotic progression of malignant cells, their clinical utility is limited by DNA repair mechanisms and immunosuppression. However, the same destructive nature of alkylation can be reciprocated at the cell surface using novel plasma membrane alkylating agents. *Results* Plasma membrane alkylating agents have elicited long term survival in mammalian models challenged with carcinomas, sarcomas, and leukemias. Further, a specialized group of plasma membrane alkylating agents known as tetra-O-acetate haloacetamido carbohydrate analogs (Tet-OAHCs) potentiates a substantial leukocyte influx at the administration and primary tumor site, indicative of a potent immune response. The effects of plasma membrane alkylating agents may be further potentiated through the use of another novel class of chemotherapeutic agents, known as dihydroxyacetone phosphate (DHAP) inhibitors, since many cancer types are known to rely on the DHAP pathway for lipid synthesis. *Conclusion* Despite these compelling data, preliminary clinical trials for plasma membrane-directed agents have yet to be considered. Therefore, this review is intended for academics and clinicians to postulate a novel approach of chemotherapy; altering critical malignant cell signaling at the plasma membrane surface through alkylation, thereby inducing irreversible changes to functions needed for cell survival.

## Introduction

Alkylation of vital intracellular nucleophiles is a nonspecific, but effective antineoplastic strategy in many hematological malignancies and some solid tumors due to subsequent perturbation of cell proliferation. Ever since Goodman, Gilman, and colleagues at Yale began investigating the potential of nitrogen mustards in 1942, alkylating the DNA of rapidly proliferating cells has been seen as a logical and highly effective method to mitigate neoplastic growth [1]. Such agents are known for covalently modifying nitrogenous bases in DNA, prompting the formation of adducts, and potentially crosslinks in the case of difunctional agents that eventually induce apoptotic signaling [2]. The success of alkylating agents has prompted the development of pseudo-alkylating agents such as cisplatin (cis-diamminedichloroplatinum(II)) and related derivatives which induce 1,2-intrastrand crosslinks with purine bases, but have no alkyl groups available for an alkylation reaction [3, 4].

As with other nonspecific cytotoxic antineoplastic agents, alkylating agents and related compounds have notable limitations. In addition to their less than ideal toxicity profile, the crosslinking capability of these agents is considerably reduced in the presence of the DNA-repair enzyme O-6-methylguanine-DNA methyltransferase (MGMT) [2, 5, 6]. Alkylating-like agents also face a similar problem of rapid DNA repair as the propensity of nucleotide excision repair (NER) is ever-present in a substantial variety of cancers. This is exemplified by non-small cell lung carcinomas (NSCLCs) which have particularly dismal prognoses due to their ability to mitigate the effects of platinum-based agents, other traditional chemotherapeutic approaches, and biologics [7–10]. Therefore, the ability of malignant cells to repair damaged DNA is an apparent constraint on the efficacy of currently approved alkylating agents. If there was a way to reciprocate the same destructive nature of alkylation in organelles besides the nucleus, high rates of apoptosis, and other forms of cell death may still be observed in the presence of DNA alkylation-resistant cells.

Such a novel chemotherapeutic approach may be attained by alkylating the plasma membrane of neoplastic cells. It has been known for quite some time that plasma membrane proteins exposed on the cell surface have important biological functions, such as cell signaling, ion transport, and cell-cell and cell-matrix adhesion interactions [11–14]. Due to recent advances in genomic, transcriptomic, and proteomic analysis, it has been elucidated that the expression level of many plasma membrane proteins is altered in malignant cells [11, 12, 15]. Such protein alterations often confer metastatic properties, creating a target for antibodies and other biologics used in the clinical setting. While such agents target specific aberrancies on the cell surface, alkylating agents could react nonspecifically with functional groups on the exterior of the plasma membrane [16], circumventing the need for a designated target to be present.

Since cell surface glycoconjugates are pivotal in surface membrane biochemistry, it is plausible that altering such signaling through alkylation could have profound chemotherapeutic activity. In fact, numerous in vitro and in vivo experiments have demonstrated the potential efficacy of plasma membrane alkylating agents that target carbohydrate moieties [17–21]. This should come as no surprise as cell surface carbohydrates are involved in multitudes of important physiological processes. Carbohydrates are involved in the adhesion of cells to substrates, as well as their adherence to each other. They have shown to change in accessibility as a function of the cell cycle, and have been indicated to play a pivotal role in cell differentiation [14, 15].

Further, cell surface carbohydrates have a profound influence on host immune response. Lectin-like carbohydrate binding sites are integral for the interaction of cytokines with their targets. Carbohydrates are also involved in the chemotaxis and extravasation of granulocytes and mononuclear agranulocytes, indicating that agents with a carbohydrate moiety may influence host immunogenicity. The humoral immune response is also markedly characterized by carbohydrate dependence, as T-lymphocytes have lectin-like carbohydrate receptors that affect antigen-specific in vitro assays [17, 18].

Despite these data, preliminary clinical trials for carbohydrate-mediated plasma membrane alkylating agents have yet to be considered. Therefore, this review is intended for academics and clinicians to postulate a novel approach to chemotherapy; altering critical malignant cell signaling at the plasma membrane surface through alkylation, thereby inducing irreversible changes to functions needed for cell survival. It is hoped that such an analysis will provide enough data to warrant further in vivo and eventual clinical trials, ultimately potentiating a new paradigm of chemotherapeutic agents.

## In vivo data provide characteristics of ideal plasma membrane alkylating agents

Many plasma membrane alkylating agents have been shown to exert significant physiological effects by chemical alteration of surface membrane electron donors, particularly at carbohydrate-specific receptor sites due to a reactive α-halo functional group, as well as their observed amphiphilic nature. In particular, halocarbonyls (haloketones, haloesters, and haloacetamides) have shown significant antitumor activity in murine models after only a single injection of an active agent [16–18]. However, simple haloacetamides and haloacetates are not active against Ehrlich ascites murine carcinoma in CD2F1 mice [17], suggesting that active halo compounds have particular physicochemical properties atypical of common halo compounds.

**Fig. 1:**
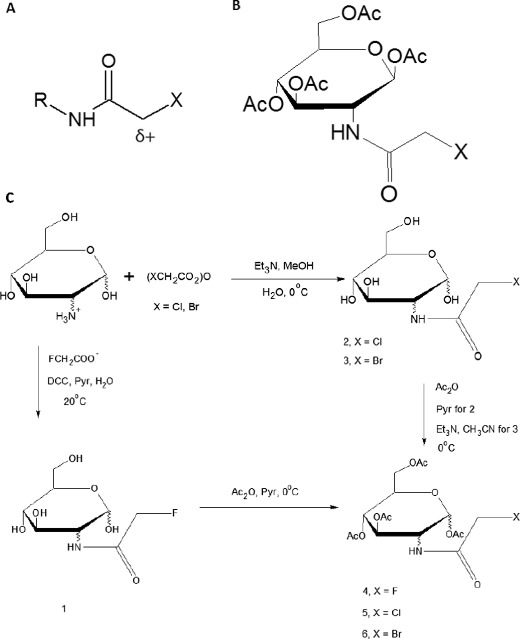
Characterization of haloacetamide structure. **a** General structure of haloacetamides. The highly reactive halogen (X) is ideal for an alkylation reaction. Such compounds are known to react by S_N_2 mechanisms, enabling a concerted reaction to occur in hydrophobic environments, such as the plasma membrane. Reactivity at the CH_2_X group is controlled by the electron withdrawing or donating power of the R group, and the corresponding pK_a_ of the parent amine. The hydrogen bond donating and accepting properties of the amide nitrogen allow the reactivity to be modulated by the dielectric constant of the in vivo environment. **b** Basic structure of haloacetamido carbohydrates. C) Synthesis of tetra-O-acetylated D-mannose analogs. A different set of reactions are needed to make the fluoro derivative compared to the bromo and chloro derivatives. Mannose analogs were devised since 2-deoxy-2-acetamido-D-mannose is a metabolic precursor for sialic acid, a vital component of cell-surface biochemistry. Panels A and B were adapted from [21]. The scheme for D-mannose analogs was adapted from [18]

Critical analysis of effective halo compounds in vivo has revealed such agents are able to partition from an aqueous to hydrophobic environment (amphiphilic log P value), and have a marked propensity to act as S_N_2 alkylators that react with strong electron donors [21]. By contrast, related compounds that are only strong alkylators, or amphiphilic do not potentiate the same antitumor activity [16–18]. Further, active haloacetamide compounds often have additional electron withdrawing groups which greatly increase alkylation potential. The pK_a_ of the parent amine from which active haloacetamide compounds are made correlates well with the alkylating activity of the antitumor agents, as is expected; the more acidic the pK_a_, the more reactive the derived α-haloacetamide should be. In fact, their reactivity at the CH_2_X group is controlled by the electron withdrawing or donating power of the R-group, and the corresponding pK_a_ of the parent amine (Fig. 1a). This also happens to be the case with nitrogen mustards which have long been used in the clinical setting due to their alkylating potential. However, haloacetamides have apparent hydrogen bond donating and accepting properties due to the presence of the amide nitrogen, indicating that compounds can be designed to have their activity modulated by the dielectric constant of the in vivo environment [21]. That localization could in turn be readily controlled by the lipid: water partition coefficient of the given haloacetamide. Further, nitrogen mustards react by S_N_1 mechanisms [22], which are not partial to hydrophobic environments; ideal for alkylating hydrophilic DNA, but not the hydrophobic environment of the plasma membrane. Due to the S_N_2 mechanisms observed in haloacetamides, the compounds react with electron donors even in nonpolar environments, allowing alkylation of functional groups on the surface of the plasma membrane to be a feasible prospect.

## Further improving specificity using acetylated carbohydrate derivatives

Experimental data comparing structural activity and chemotherapeutic effectiveness have confirmed that halo carbonyls act as non-charged hydrophobic or amphiphilic electrophiles. Further, the compounds with the most antitumor activity in these series are strong alkylating agents. Since antitumor activity is dependent on the polarity and alkylating activity of halo carbonyls, it has been postulated, and later confirmed that such analogs exert their effects by alkylating cell surface nucleophiles. To further improve the specificity of haloacetamides, the R group has been substituted with carbohydrate derivatives that are found on the plasma membrane. However, inserting a hydrophilic moiety into the base structure significantly reduces antitumor activity, as observed by haloacetamides with free sugars [17, 18]. Therefore, any carbohydrate structure inserted into the haloacetamide base structure needs to be altered so that the resulting compound retains its affinity for nonpolar environments.

One approach to circumvent this issue has been to acetylate the carbohydrate derivatives with acetic anhydride after being attached to the haloacetamide (Fig. 1b and c). From this approach, a comprehensive series of tetra-O-acetate haloacetamido carbohydrate analogs (Tet-OAHCs) have been developed to mimic carbohydrates used at the plasma membrane surface, including 2-deoxy-2-acetamido-β-D-glucose and its α anomer, 2-deoxy-2-acetamido-β-D-galactose, and 2-deoxy-2-acetamido-D-mannose. The mannose derivative is particularly important, as it is a metabolic precursor of sialic acid, an integral component of cell surface biochemistry [23–25]. In fact, metastatic cancer cells often express a high density of sialic acid-rich glycoproteins, creating a negative charge on cell membranes [26, 27]. The subsequent repulsion between adjacent malignant cells is pivotal for the metastasis of many late stage cancers, providing an optimal target for haloacetamido mannose analogs.

The importance of the hexose tetra-O-acetate carbohydrate moiety for haloacetamides has become apparent in the treatment of tumor-bearing mice. Several compounds in the glucosamine, galactosamine, and mannosamine series have produced considerable rates of long term survival with almost all mice surviving an intraperitoneal (i.p.) injection of 2.5 × 10^7^ Ehrlich ascites carcinoma cells, a concentration that is ten times the minimum dose lethal to 95 % of B6D2F1 mice [21]. By contrast, simple haloacetamide and haloacetyl compounds were completely inactive in the tumor-bearing mammalian system [21]. Further, non-hexose bromoacetamido polyol acetates with alkylating activities and log P values spanning those of the active bromoacetamido hexose tetra-O-acetates did not exhibit antitumor activity [21]. Therefore, the chemotherapeutic potential of Tet-OAHCs requires more than the physicochemical properties of alkylating activity and the octanol: water partition coefficient, as it is associated with the specific interaction these agents can confer with functional groups on the surface of the plasma membrane.

## Tetra-O-acetylated haloacetamido carbohydrate analogs potentiate leukocyte influx

It is has been well-established that carbohydrates are pivotal for leukocyte function. O-linked glycans of glycosylation-dependent cell adhesion molecule-1 (GLYCAM-1) are used by leukocytes to home in on sites of inflammation, as well as direct lymphocytes to lymph nodes [28]. Further, carbohydrates are critical for diapedesis, an innate form of extravasation in which carbohydrate ligands on the circulating leukocytes bind selectin molecules on the inner wall of the vessel, enabling the blood cells to slow down and begin rolling along the inner surface of the vessel wall until they have passed through the vascularized tissue [29, 30]. Therefore, it seems plausible that carbohydrate-based chemotherapeutic agents could have a profound influence on leukocyte influx at the site of administration.

When N-bromoacetyl-β-D-glucosamine tetra-O-acetate (NBrAcGlc-TA) was shown to produce consistent long term remission in Ehrlich tumor-bearing mice after a single injection (0.11 mmol/kg), it was observed that the sites of tumor challenge and drug administration had a rapid granulocyte influx [21]. Further, N-bromoacetyl-β-D-galactosamine tetra-O-acetate (NBrAcGal-TA) injected i.p. at the same dose one day prior to a challenge of 1 × 10^6^ Ehrlich ascites carcinoma cells conferred substantial resistance to lethal tumor outgrowth when compared to vehicle-treated control animals. Although plasma membrane alkylating chlorohydroxyacetone-benzoate derivatives were shown to produce long term survival in the same tumor system [31], they were not associated with an increased granulocyte influx, nor did pretreatment of such compounds one day prior to tumor challenge confer any noticeable resistance.

To further examine whether Tet-OAHCs potentiate a substantial in vivo immune response, NBrAcGlc-TA and chlorohydroxyacetone dinitrobenzoate (ClHA-DNB) were injected at their respective effective doses in B6D2F1 mice in the absence of tumor challenge [31]. Results from the study indicated that only the carbohydrate analog produced a marked early increase in granulocyte influx, as well as a pronounced increase in later lymphocyte response. A quantitative comparison of host leukocyte influx between carbohydrate analogs and another hydrophobic monofunctional non-carbohydrate alkylating agent, bromohydroxyacetone benzoate (BrHAB), later confirmed the in vivo observations [21]. It is worth noting that a difunctional alkylating nitrogen mustard was not able to confer any protection to tumor challenge when administered prior to the injection, and significantly reduced host resistance against Ehrlich ascites carcinoma due to its pronounced immunosuppressive effects [21].

## Efficacy in other cancer models

Although most studies investigating the chemotherapeutic efficacy of plasma membrane alkylating agents have been done with Ehrlich ascites carcinoma, the compounds have shown promise in other cancer models as well. In particular, chlorohydroxyacetone esters were shown to produce long term survival in mice challenged with murine sarcoma 180 and P815 murine mastocytoma [31]. However, plasma membrane alkylating agents have demonstrated substantial efficacy in disseminated hematological malignancies as well. Tet-OAHCs were first examined in vitro against Friend murine erythroleukemia cells [17]. Results from the study indicated that the malignant cells in log phase suspension culture were substantially inhibited by low concentrations of bromoacetamido and chloroacetamido analogs of the glucose and galactose series.

The success of plasma membrane alkylating agents against Friend erythroleukemia in vitro prompted an in vivo study examining the effects of such compounds on the immunogenicity of the malignant cells. When DBA/2 J mice treated with chlorohydroxyacetone benzoate (ClHAB) survived a primary challenge of a single injection of 1 × 10^6^ tumorigenic cells, the survivors were completely resistant to rechallenge. By contrast, a single injection of sonically disrupted Friend erythroleukemia cells or of virus harvested from cultured cells had no effect on the survival time of animals subsequently rechallenged with intact cells, indicating that ClHAB potentiates an immunogenic response. Further, mice given injections of cells killed by exposure to 50 μM ClHAB in vitro exhibited increased survival times upon rechallenge. It was later demonstrated that Friend erythroleukemia cells treated for 3 h with 30 μM ClHAB were non-proliferative in subculture, and completely non-tumorigenic when implanted either i.p. or subcutaneously (s.c.) into DBA/2 J hosts. In fact, mice receiving a single injection of 1 × 10^6^ cells altered under these conditions were fully protected against rechallenge. These results are indicative of substantial reductions in tumorigenicity, as it is known that as few as 10 cells of Friend erythroleukemia are needed to kill murine models [32, 33]. Therefore, controlled modification of Friend erythroleukemia cells by exposure to relatively low concentrations of ClHAB abrogated the tumorigenicity of treated cells, without altering their ability to confer protection to subsequent rechallenge with unaltered cells.

## Potentiating activity by exploiting differences in phospholipid metabolism

Although carbohydrate analogs offer a potentially specified approach to alkylate functional groups vital for neoplastic cell stability, it is likely that some cells within various cancer types will have inherent or acquired resistance to the series of compounds. Therefore, finding additional agents that can supplement the mechanisms of plasma membrane alkylating agents is clinically relevant, as it will give clinicians a greater diversity of options in which to treat problematic tumorigenic growths.

NAD-linked glycerol-3-phosphate dehydrogenase (GPDH) is a key enzyme linking carbohydrate metabolism with the synthesis and degradation of glycerolipids, yet it is present in low amounts or entirely absent in most neoplastic tissues [34–36]. Since phospholipids are required for the synthesis of new membranes in proliferating cells, absence of this enzyme in malignant cells is profound, and indicates that alternative pathways to glycerolipids are pivotal for the potentiation of neoplastic tissue. Such an alternative pathway exists in which dihydroxyacetone phosphate (DHAP) is acylated and reduced to lysophosphatidic acid by a microsomal NADP-linked enzyme entirely independent of cytosolic GPDH [37–40]. The acyl-DHAP pathway results in phospholipid synthesis, as well as production of ether lipids that are often elevated in various cancer types, indicating that such compounds influence the abnormal growth patterns of these malignancies [35]. Therefore, DHAP analogs designed to exploit the aberrant activity of GPDH could profoundly interfere with lipid metabolism and membrane glycerol-lipid synthesis, making affected cells much more sensitive to chemotherapeutic agents that directly attack the plasma membrane.

Attempts have been made to exploit these potential differences and to alter membrane-mediated phenomena in neoplastic tissue directly at the level of membrane lipids, as well as indirectly by affecting lipid metabolic pathways. Success has been achieved with halo analogs of GPDH and DHAP [41–43] which are designed to exploit the differences in GPDH production between malignant and normal cells. The glycerol analogs directly exploit the difference in GPDH, while the DHAP analogs influence the GPDH and acyl-DHAP pathways. These analogs have shown some in vivo antitumor activity, but they experience difficulties traversing the plasma membrane, as they are charged, phosphorylated compounds [44]. Therefore, non-phosphorylated derivatives of dihydroxyacetone may be pivotal for increasing chemotherapeutic activity. This reasoning is in alignment with previous observations that monobenzoate esters of dihydroxyacetone inhibit the acyl-DHAP pathway. However, the inhibitory effect of monobenzoate esters is substantially limited in vivo by endogenous kinase activity which phosphorylates at the 1′ position of the compounds, resulting in the formation of 1,3-DHA-monobenzoate, a non-inhibitory derivative [45]. Since non-phosphorylated halo derivatives of dihydroxyacetone are not susceptible to kinase inactivation, they could be used as potent inhibitors of the acyl-DHAP pathway.

## Novel compounds worth investigating for chemotherapeutic potential

**Fig. 2:**
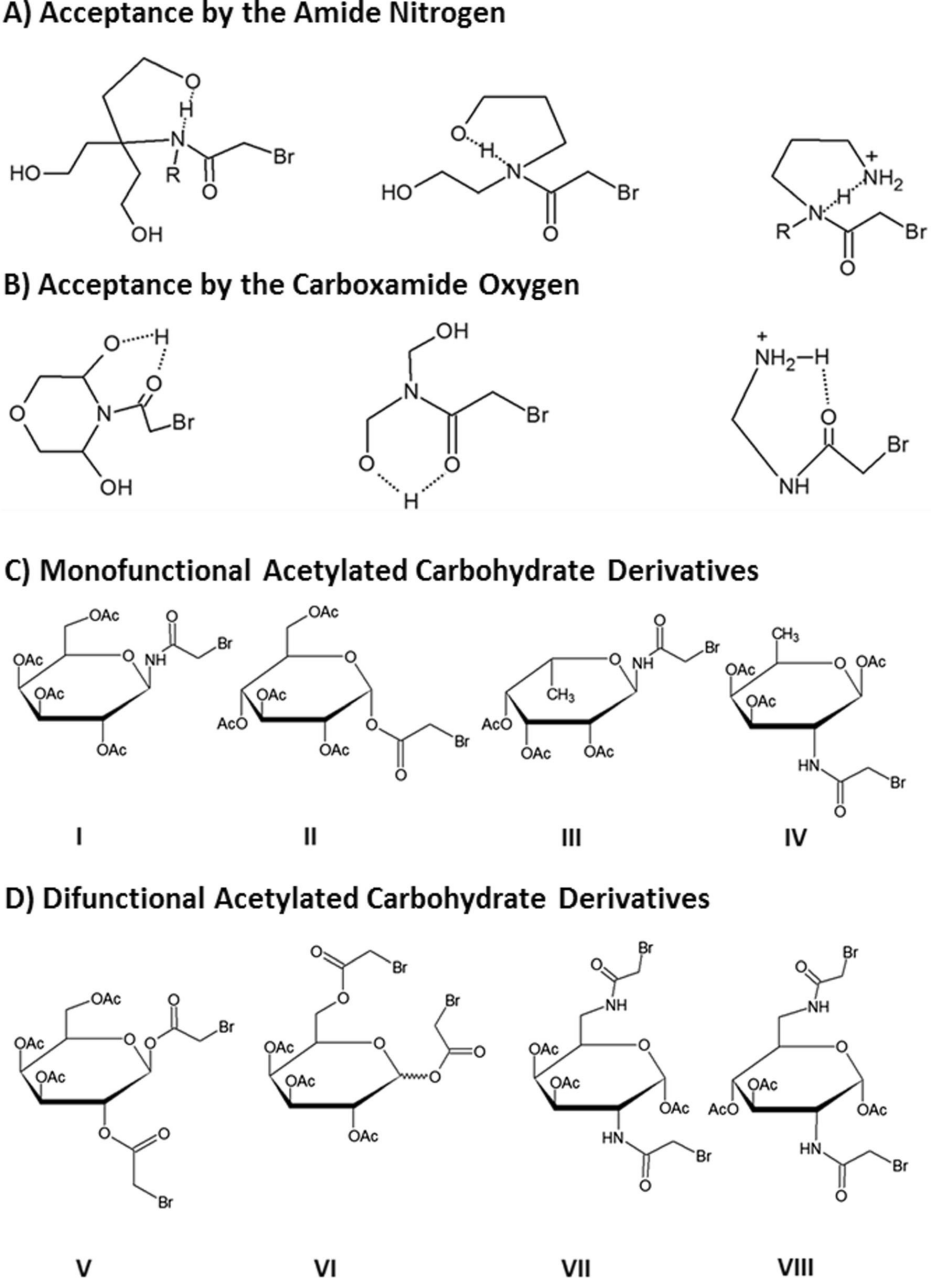
Proposed compounds to enhance alkylation activity through intramolecular hydrogen bonding and novel acetylated carbohydrate analogs for preclinical evaluation. **a** Compounds that form a hydrogen bond from acceptance by an amide nitrogen. **b** Compounds that confer the same potential through hydrogen bond acceptance by a carboxamide oxygen. Formation of ring forming intramolecular hydrogen bonding is highly favorable in a medium of a low dielectric constant, typically observed in low polarity environments. **c** Monofunctional derivatives are as follows: I) N-bromoacetyl-galactosylamine-2,3,4,6-tetra-O-acetate, II) 1-O-bromoacetyl-glucose-2,3,4,6-tetra-O-acetate, III) N-bromoacetyl-fucosylamine-2,3,4-tri-O-acetate, and IV) 6-deoxy-N-bromoacetyl-galactosamine-1,3,4-tri-O-acetate. D) Difunctional derivatives are as follows: V) 1,2-di-O-bromoacetyl-β-D-galactose-3,4,6-tri-O-acetate, VI) 1,6-di-O-bromoacetyl-α or β-D-galactose-2,3,4-tri-O-acetate, VII) 2,6-di-N-bromoacetyl-2,6-dideoxy-2,6-diamino-galactose-1,3,4-tri-O-acetate, and VIII) 2,6-di-N-bromoacetyl-2,6-dideoxy-2,6-diamino-β-D-glucose-1,3,4-tri-O-acetate

Although a considerably diverse set of plasma membrane alkylating agents have been examined for chemotherapeutic potential, they are only preliminary investigations in what could turn out to be a large class of similarly designed chemotherapeutic agents. Plasma membrane alkylating agents are only in their initial stages of development, and many more compounds can be designed and studied for anticancer activity. For example, it has been observed that haloacetamide compounds that exhibit alkylating potential have their reaction rate dictated by hydrogen bonding [17]. While hydrogen bond accepting by C = O, C-O, and C-N structures accelerate the rate of alkylation, hydrogen bond donation by N-H decreases observed activity. It would therefore seem apparent that agents without a hydrogen on the amide nitrogen could be particularly effective alkylators. Further, since hydrogen bonding can be exploited as a means to enhance alkylation in a hydrophobic environment, compounds should be designed to examine potential therapeutic benefits conferred from such activity. Pragmatic requirements to observe hydrogen bond controlled enhancement of alkylation by haloacetamides in low polarity medium are as follows: 1) Hydrogen bond donation by N-H is an effective gain of an electron by N, indicating that the alkylation rate should decrease; 2) Hydrogen bond acceptance by N-H or C = O is an effective withdrawal of an electron, inherent for acceleration of the alkylation rate; 3) Intramolecular hydrogen bonds forming 5 and 6-membered rings (including the shared hydrogen) would be highly favored due to the stabilization of ring formation; 4) Formation of ring forming intramolecular hydrogen bonding would be favored in a medium of a low dielectric constant (such as the plasma membrane) compared to a polar medium; 5) Multiple intramolecular hydrogen bonds would be more effective than solvent donated hydrogen bonding in enhancing alkylation rate; 6) Any intramolecular hydrogen bonding should significantly enhance the rate of alkylation. Due the potential importance intramolecular hydrogen bonding could have in increasing plasma membrane alkylating activity, several novel compounds have been posited for chemotherapeutic investigation (Fig. 2a and b).

One of the most profound characteristics of plasma membrane alkylating haloacetamides is their ability to potentiate a substantial granulocyte influx at the drug administration and primary tumor site when attached to a tetra-O-acetylated carbohydrate. Not only does administration of such compounds 24 h after tumor challenge result in particularly high cure rates and resistance to rechallenges, but pretreating mice with the congeners effectively prevents tumor formation. Although consistent alkylation of the plasma membrane is enough to warrant further investigation due to the importance of cell surface biochemistry in neoplastic growth, the ability to potentiate an immune response at the primary tumor site has notable clinical implications, and further substantiates the utility of acetylated carbohydrate haloacetamide analogs. It is apparent that these compounds could have immediate therapeutic potential, and novel congeners have been proposed to further examine this intriguing in vivo response (Fig. 2c and d). The compounds utilize analogs of carbohydrates typically observed on the cell surface to promote alkylating specificity. In addition to monofunctional carbohydrate derivatives, difunctional haloacetamide compounds are also posited as a means to further increase alkylation rates. It is also well known that difunctional alkylating agents have the propensity to crosslink adjacent nucleophiles [1], and in theory should give haloacetamido analogs another mechanism by which to damage neoplastic cells.

## Potential synergy with anthracyclines

Although this review has commented on novel plasma membrane-directed agents, there is a class of clinically approved antineoplastic agents that has a marked influence at the cell surface. Anthracyclines are most commonly known as nucleic acid-directed agents, as the compounds intercalate base pairs, produce free radicals, and are potent inhibitors of DNA topoisomerase II [4]. However, it has also been well established that anthracyclines (particularly doxorubicin) alter the fluidity of neoplastic cell plasma membranes [46, 47], and bind phospholipids with considerably affinity [48, 49]. Some studies have also indicated that extracellular doxorubicin is important for anticancer activity and that the compound demonstrates marked cytotoxicity without entering the cell [4, 50].

While the combination of anthracyclines and plasma membrane alkylating agents has yet to be assessed for drug synergy, this concomitant chemotherapeutic approach is an intriguing possibility that would give these novel agents more versatility if they ever reached the clinical setting. Indeed, anthracyclines and DNA-directed alkylating agents are often used in combination, with synergy being attributed to the production of free radicals and alkylated adducts. Interestingly, it has been demonstrated that doxorubicin produces free radicals, including highly reactive hydroxyl species, at the cell surface. Therefore, it is possible that the same synergy between anthracyclines and DNA-directed alkylating agents may be reciprocated with plasma membrane alkylating agents, due to the combined effect of unstable free radicals and alkylated adducts. However the potential cardiotoxicity of plasma membrane directed agents has also yet to be assessed, and would likely decide whether these agents could be used concomitantly with anthracyclines, unless dexrazoxane or other cardiotoxic reducing agents mitigate any substantial aberrant drug interaction.

## Conclusion

**Fig. 3:**
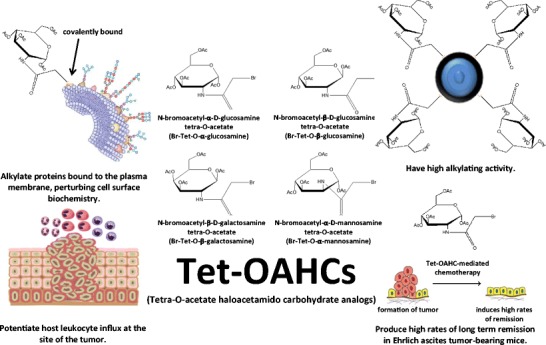
Antineoplastic mechanisms of tetra-O-acetate haloacetamido carbohydrate analogs

Plasma membrane alkylating agents are a novel class of antineoplastic agents that have the potential to supplement current chemotherapeutic protocols. Unlike traditional alkylating agents, compounds that have an affinity for the cell surface do not need to gain entry into the cellular interior, and therefore circumvent drug efflux mechanism of various cancer types. In effect, such compounds offer a novel target for clinicians to exploit in combinatorial chemotherapy that could be used concurrently with DNA-directed alkylating agents, as well as drugs of other mechanisms. Tet-OAHCs are particularly interesting carbohydrate-based alkylating agents, as they potentiate a substantial granulocyte influx at the administration and primary tumor site in addition to having high alkylating activity in amphiphilic environments that together potentiate sustained tumor regression in mammalian models of malignancy (Fig. 3). This is a particularly important observation, as traditional DNA-directed alkylating agents often have aberrant side effects on the patient’s immune response [4, 51, 52]. An antineoplastic agent that promotes leukocyte influx could be a particularly beneficial supplement to many current chemotherapeutic protocols, and warrants further examination of these novel congeners.

However, there will inevitably be some neoplastic cells resistant to the initial effects of plasma membrane alkylating agents, which is why indirectly targeting the plasma membrane of malignant cells through the use of halo and nonphosphorylated analogs of DHAP in concurrence with cell surface alkylation appears to be a sensible prospect. Unlike normal cells, malignant cells often rely on the acyl-DHAP pathway, as they have significantly depleted levels of GPDH. Antineoplastic analogs of DHAP cripple the acyl-DHAP pathway, leaving neoplastic cells very few options for lipid synthesis. With an aberrant plasma membrane already present, further perturbation with cell surface-directed alkylating agents should potently inhibit many cancer types.

Although the plasma membrane is a relatively new therapeutic target, it may prove to be an effective method of acquiring preferential damage through the introduction of novel antineoplastic agents such as plasma membrane alkylators and acyl-DHAP inhibitors. Since these compounds offer relatively novel mechanisms of action, more in vivo studies, followed by numerous clinical trials will be needed to accurately assess whether plasma membrane-directed agents actually have therapeutic efficacy. If plasma membrane alkylating agents and DHAP analogs do prove to have clinical relevance, they could be used concomitantly with currently approved chemotherapeutic approaches to increase the efficacy of such protocols. Needless to say, this will only occur through further in vivo observation. Only through such measures will enough data be acquired to definitively determine whether plasma membrane-directed agents have significant clinical relevance.
